# Impact of French lockdowns on bereavement experiences: Insight from ALCESTE analysis revealing psychological resilience and distinct grief dynamics amidst COVID‐19

**DOI:** 10.1002/ijop.13267

**Published:** 2024-11-19

**Authors:** Livia Sani, Yasmine Chemrouk, Boris Lassagne, Chad Cape, Marie Ngo Nkana, Jacques Cherblanc, Marie‐Frédérique Bacqué

**Affiliations:** ^1^ SULISOM UR 3071 Université de Strasbourg Strasbourg France; ^2^ Clinique Psychanalyse Développement ‐ EA 4430 Université Paris Nanterre Nanterre France; ^3^ Human and Social Sciences Université du Québec à Chicoutimi (UQAC) Saguenay QC Canada

**Keywords:** COVID‐19, Lockdown, Grief, ALCESTE, Loss

## Abstract

At the beginning of 2020, the entire world was shocked by a global health emergency. According to the literature, fear, high mortality and health restrictions had significant psychological consequences on the population. This study evaluates the French lockdown's impact on the grieving process and how people worked through their grief. Two semi‐structured interviews were conducted with 31 participants who had lost a loved one between March 2020, June, and September 2021 (T0) and 6 months later (T1). Subsequently, they were divided into two groups: those who lost someone during the first lockdown (Group 1) and those who lost someone outside the lockdown periods (Group 2). The interviews were analysed using the ALCESTE software, a statistical analysis tool for textual data based on word co‐occurrences. This research significantly advances the understanding of bereavement during crises, providing new perspectives and practical insights for policymakers, healthcare professionals and support organisations. Its methodological innovation and detailed analysis contribute to the ongoing discussion on grief and resilience in challenging circumstances. Ultimately, this study lays the foundation for improved support and intervention strategies tailored to the needs of bereaved individuals during crises.

The onset of 2020 marked the emergence of the SARS‐CoV‐2 virus, which rapidly evolved from an unknown threat into a global pandemic, resulting in a tragic toll of 6,319,395 deaths worldwide (Coronavirus, 2023). In response to this public health emergency, governments worldwide implemented various strategies to curb the risk of contagion, including lockdowns and restrictions on travel and migration from high‐risk countries.

Among the affected countries is France, which has suffered significant loss and is ranked 11th globally, with a total of 159,000 victims (Coronavirus, 2023).

This study focuses specifically on the French context, aiming to examine the challenges faced by bereaved individuals during the pandemic particularly in light of the measures implemented during the initial lockdown.

The French government responded to the emergency with stringent containment measures, including stay‐at‐home orders, travel limitations and bans on social gatherings.

The first lockdown period, lasting from March 17th to May 11th, 2020, markedly disrupted French citizens' social fabric and daily routines for 55 days (Declaration by Mr. Emmanuel Macron, 2020). During this time, the majority of the French workforce—except for essential workers such as healthcare professionals and law enforcement—was either forced to transition to remote work or suspend their professional activities (Declaration by Mr. Emmanuel Macron, 2020).

Following the initial lockdown, France experienced subsequent lockdowns (30 October 2020 to 15 December 2020; 3 April 2021 to 3 May 2021; 30 October 2021 to 14 December 2021) with less stringent restrictions. These later measures included more lenient confinement rules, the reopening of economic activities, relaxed regulations on social gatherings, fewer travel restrictions and improved access to essential services.

However, implementation of social distancing measures drastically reduced social interactions and impacted the quality of personal relationships, leading to a significant increase in depressive symptoms, anxiety, mood and sleep disorders, post‐traumatic stress disorder (PTSD) and cognitive decline over time (TMGH‐Global COVID‐19 Collaborative, 2021). These effects had a disproportionate impact on young adults (16–24 years), the elderly, and individuals with pre‐existing psychiatric diagnoses such as anxiety and depression (Gandré et al., 2020; Hwang et al., 2020).

Furthermore, stress levels during the period of social isolation, defined as the objective state of individuals' social environments and interactional patterns (Hwang et al., 2020), were significantly exacerbated by various socio‐demographic factors, including marital status, female gender, low education level and confirmation of COVID‐19 contraction (Gandré et al., 2020; Hwang et al., 2020; TMGH‐Global COVID‐19 Collaborative, 2021). It is worth noting that the national context further shaped the association between social and psychological stress. For instance, countries with higher mortality rates, less restrictive policies, weaker state capacities or greater globalisation recorded higher stress levels associated with social isolation, while those with more stringent or effective containment measures mitigated these adverse effects (Buecker et al., 2021; Buecker & Horstmann, 2021).

Additionally, among the psychological challenges posed by social isolation were the restrictions on traditional funeral practices, which deprived individuals of the customary rituals and support systems crucial to the grieving process (Dew et al., 2022; Hanna et al., 2021).

During the first lockdown, the French population faced bereavement without the ability to be present with their sick loved ones in their final moments, participate in traditional mourning rituals or engage in communal ceremonies, such as body washing (or *toilette funéraire* in French), wake attendance, cemetery accompaniment or ceremonies with more than 30 people.

Research has demonstrated that losing someone during the pandemic significantly increased the risk of developing PTSD and complicated (or prolonged) grief disorder, even among children. For instance, in Quebec, out of 955 study participants, 15% received a provisional diagnosis of prolonged grief disorder (Cherblanc et al., 2021). Prolonged grief disorder is characterised by a persistent and pervasive longing for the deceased and/or cognitive preoccupation with the deceased, combined with intense emotional pain, disbelief, isolation, avoidance of places or situations, loss of interest and anger for at least 6 months after the loss (Dew et al., 2022; Prigerson et al., 2009; Shear, 2015).

Despite existing research investigating the overall impact of the pandemic on mental and social health, the specific experiences of bereaved individuals during the lockdown remain poorly understood. This study aims to address this gap by examining the unique challenges encountered by individuals affected by loss during the COVID‐19 pandemic in France. Through qualitative analysis, we intend to explore the interaction between social isolation and grief experiences, thereby providing valuable insight into the underlying mechanisms of grief during a global health crisis. In particular, we aim to investigate potential differences in grief experiences between the initial strict lockdown period and subsequent phases of the pandemic. To achieve this, a hypothesis and research question have been formulated:

The lockdown and its regulations have specifically impacted the grieving process.

What are the different experiences and emotions that emerge between those who lost someone during the lockdown compared with those who were not subjected to such strict restrictions?

## METHODS

This study is an integral part of COVIDEUIL, a retrospective mixed longitudinal research project on the restrictions on funeral rites that began in France but is now being conducted internationally in Italy, Switzerland, Belgium (Boever et al., 2023), Greece (Koliouli et al., 2024), Quebec (Cherblanc, Simard, et al., 2022; Cherblanc, Zech, et al., 2022) and Mexico.

The data presented below have been extracted from those of COVIDEUIL‐France, with particular reference to the psychological effects that the lockdown may have produced on bereaved people.

### Participants

Thirty‐one participants were recruited from April to September 2021 by posting an advertisement on social media pages dedicated to bereavement and/or COVID‐19 (Facebook and LinkedIn), and through French support groups for bereaved people, some related to COVID‐19 (i.e., *Vivre son deuil, CoeurVide19, Une rose blanche, Victimes du covid 19, Empreintes*), and newspapers (*Le Journal de la Haute‐Marne, La République du Centre*).

Only French speakers of legal age with no cognitive disorders and who had lost someone after March 2020 could participate in the study. The sample was then divided into two groups: those who lost someone during the first lockdown (11 people, Group 1) and those who lost someone during the pandemic but at a time when there was no lockdown and the general restrictions were less strict (20 people, Group 2).

As Table 1 shows, the majority of participants were women (91% in Group 1; 75% in Group 2), with an average age of 53.5 years in Group 1 and 46.1 years in Group 2. In terms of relationship status, 64% of Group 1 participants and 40% of Group 2 participants were living with a partner.

**TABLE 1:** Socio‐demographic characteristics of the sample

	Groups	
	Group 1 (*n* = 11)	Group 2 (*n* = 20)
Gender, *n* (%)		
Participants		
Male	1 (9)	4 (20)
Female	10 (91)	15 (75)
Non‐binary	0	1 (5)
Deceased		
Male	7 (64)	12 (60)
Female	4 (36)	8 (40)
Age, mean (*SD*)		
Participants	53.5 (12.7)	46.1 (16.0)
Deceased	70.5 (19.9)	71.1 (17.4)
Marital status, *n* (%)		
Divorced	1 (9)	0
Single	0	7 (35)
Widowed	2 (18)	5 (25)
With a partner	8 (73)	8 (40)
Living alone, *n* (%)		
Yes	4 (36)	12 (60)
No	7 (64)	8 (40)
Who was the deceased, *n* (%)		
Partner	2 (18)	5 (25)
Parent	4 (36)	11 (55)
Child	1 (9)	1 (5)
Grandparent	1 (9)	2 (10)
Other relative (uncle, brother‐in‐law, etc.)	1 (9)	1 (5)
Friend	2 (18)	0
Cause of death, *n* (%)		
Cancer	5 (45)	7 (35)
COVID‐19	4 (36)	3 (15)
Suicide	0	1 (5)
Unknown	0	1 (5)
Others (cerebrovascular disease, heart disease, Parkinson's, natural death)	2 (18)	8 (40)

*Note*: Group 1 = those who lost someone during the first French lockdown; Group 2 = those who lost someone when there was no lockdown in France.

The deceased were mostly men, with an average age of around 70 years old. The causes of death are quite different between the two groups: 45% of Group 1 lost a loved one to cancer, followed by COVID‐19 (36%) and other causes (18%); in Group 2; however, the leading causes of death are other causes (40%), cancer (35%) and COVID‐19 (15%).

Most participants in both groups lost their parents (36% in Group 1 and 55% in Group 2), followed by the loss of a partner or friend for Group 1 (18%) and the loss of a partner for Group 2 (25%).

### Procedure

Participants were interviewed for the first time between June and September 2021 (T0) and subsequently 6 months afterward (T1) through semi‐structured interviews conducted in person or online via the BBB, the University of Strasbourg's communication platform; the average duration of the interviews was 49 minutes in T0 and 43 minutes in T1.

Table 2 shows the interview guide used by the researchers/psychologists who collaborated in the COVIDEUIL‐France study.

**TABLE 2:** Examples of questions from the guide used to conduct semi‐structured interviews

Categories	Examples of questions
The deceased	*Can you tell me about the deceased?* *Who was she/he?*
The death	*How did the death occur?* *How did you find out about his/her death?*
Funeral rituals	*How did the funeral rituals take place?* *How did Covid restrictions affect the rituals?* *If there had been no restrictions, how would you have proceeded?*
Grief reactions and experience Alternative and digital rites	*How did you or are you coping with your grief?* *What are the effects of this death on your life?* *What emotions have you experienced?* *Do you receive support from your loved ones?* *Have you performed or participated in alternative rites created to commemorate the deceased, such as digital rites (*e.g.*, a commemorative page on the Internet, virtual cemetery, virtual funeral, etc.)?*

The interviewers, researchers with backgrounds in psychotherapy and psychoanalysis, brought extensive experience working with individuals in mourning and the medical field. Their expertise guided the formulation of questions aimed at capturing nuanced emotional and psychological responses to loss during the pandemic, contributing to the study's specificity and aligning with broader research on bereavement dynamics during pandemics.

Furthermore, interviewers, as expert psychologists specialising in bereavement, underwent rigorous training to handle sensitive situations. They were prepared to provide resources and refer participants to professional colleagues if needed. This meticulous attention to participants' well‐being ensured a respectful interview environment that met individual emotional needs.

### Data analysis

All the collaborators of COVIDEUIL‐France transcribed interviews using the NVivo Transcription software (see acknowledgments) and verified them by the first two authors. The transcriptions were then analysed using ALCESTE (“Analyse Lexicale par Contexte dun Ensemble de Segments de Texte”, *Lexical Analysis by Context of a Set of Text Segments*), software that allows quantitative analysis of textual data (Reinert, 1987).

The number of participants was decided based on the scientific literature on qualitative analysis validity (Dworkin, 2012; Guest et al., 2006). Although the sample size may appear limited, ALCESTE's robust statistical analysis can reveal significant themes even in smaller samples by focusing on co‐occurrences of terms within the text.

First designed in 1979 by Max Reinert at Jean‐Paul Benzécri laboratory (CNRS, Paris), ALCESTE separates the text into Units of Context (UC), which are formed of one or several consecutive sentences based on the punctuation and the number of significant words.

ALCESTE evaluates the co‐occurrences in the text, that is, the frequency and position of a word, using chi‐square analysis, and then extrapolates and provides classes of words that appeared in the text, presenting them in descending order of co‐occurrence. The words and selected sentence extracts within each class are also presented according to a hierarchical classification of the co‐occurrences.

The selected and analysed extracts of sentences from the text (see Appendices S1–S3) may start or end in the middle of a sentence or correspond to a whole sentence. It is then the researcher's responsibility to identify the theme in each class. In this study, the thematic analysis was carried out by two authors (L.S., Y.C.), first independently and then together to agree on the category of each class. This dual analysis ensured a higher level of consistency and reliability in theme identification. A sample table of the various word classes identified by ALCESTE, translated into English, is provided in Appendix S3.

The analysis with ALCESTE was considered the best option for several reasons. Firstly, using ALCESTE, lexical classes and themes could be quickly and easily extracted from a large corpus (such as in this study). Furthermore, numerous studies have confirmed its scientific validity in the fields of human sciences (Geka & Dargentas, 2010; Lahlou, 1998; Metz et al., 2019; Scharnitzky & Kalampalikis, 2007), and in research specifically focused on COVID‐19 (Aperribai et al., 2020; de los Valadez et al., 2020; Idoiaga et al., 2020; Maillot et al., 2022).

Reviewing previous studies that successfully used ALCESTE to analyse similar textual data allowed for careful consideration and evaluation of its reliability, emphasising the consistency and replicability of the outcomes obtained. In addition, multiple independent researchers' analyses helped ensure the consistency of interpretations and word classes identified.

Finally, the analysis using ALCESTE was applied purposefully and consciously, considering the specificities of our text corpus, including the clear definition of UCs and careful selection of meaningful words. This attention to detail aims to ensure that the identified word classes accurately reflect the dynamics of grief during the pandemic, minimising possible bias.

The variations in thematic classes between different groups and time points reflect the dynamic nature of language and discourse classification shifts due to changes in the way participants discuss these themes over time. ALCESTE's strength lies in its capture these shifts, providing a nuanced understanding of the evolving discourse.

ALCESTE notes lexical co‐occurrences, which can vary depending on the context and the group of participants. This could potentially lead to different classifications of themes between groups and over time.

By focusing on co‐occurrences rather than just the frequency of individual words, ALCESTE reveals deeper patterns within the text, making it a powerful tool for analysing complex qualitative data, even with relatively small sample sizes.

## RESULTS

Table 3 shows the different thematic categories obtained for each of the two groups in T0 (June–September 2021) and T1 (6 months later).

**TABLE 3:** Thematic categories were obtained from the ALCESTE analysis for Group 1 and Group 2 in T0 and T1

	Group 1		Group 2	
	T0	T1	T0	T1
Class 1	Funeral restrictions	End‐of‐life context	End‐of‐life context	Social media
		Health restrictions (hospital context)	COVID‐19	Current grief
				Funerals rites
Class 2	Medical and healthcare team	Funeral restrictions	Grief experience	End‐of‐life context
	End‐of‐life context	Other arrangements	Emotions of loss	Health restrictions
			Social support	
Class 3	Grief experience	Current grief	Funeral rites	Seeing or touching the body
Class 4	Social support	Memories of the deceased		
	Other bereavements	Social support		

ALCESTE identified four classes of words for Group 1, those who lost someone during the first lockdown, and three classes for Group 2, those who lost someone during the pandemic but not during a lockdown (and therefore without strict general restrictions).

In the appendices, one would find the sentences selected and analysed by ALCESTE for Groups 1 and 2 (see Appendices S1 and S2), which correspond to the first five to six excerpts with the highest chi‐square.

As time passes and the grieving process continues, certain themes may become more prevalent in participants' narratives, which could explain why certain thematic classes emerge more clearly at different time points or in different clusters.

### Thematic analysis and comparison of Groups 1 and 2 in T0

#### Funeral restrictions

For Group 1, this category concerns the participants' perception regarding the regulations put in place during the pandemic. For example, how, in following such rules, professionals handled the body and coffin of the deceased:

There was no preparation of the body, so we had the impression that he was put in a garbage bag like a plague victim and then put in a coffin. A few days later, we found ourselves in front of a closed coffin…We put a photo on the coffin to know it was him.

The term “bag” or “trash bag” appeared in most interviews. In addition, the interview excerpts revealed deep feelings of anger, misunderstanding, and bewilderment. Group 2 presents *Funeral rites*, in which various elements concerning the funeral are described, such as the church, flowers, service, and the cemetery.

Health restrictions are rarely mentioned, and when they are, they do not seem to have hindered the desired course of the funeral. Thus, the funeral rites are evoked from the “ordinary” point of view of the bereaved, reflecting customary practices and how each person was able to take advantage of the funeral (whether religious or not) to pay tribute to the deceased.

We had both a religious ceremony and a cemetery ceremony. She had a beautiful ceremony, everyone told us. I forced myself and wrote a text, I struggled with myself to read it out.

#### End‐of‐life context and hospital environment

For Group 1 we can identify two categories related to these themes: the first one is *Medical and healthcare team*, which concerns the relationship and communication with doctors and nurses; the second theme concerns *end‐of‐life context*, that is, disease and medical intervention. Participants discussed their relationships with professional caregivers and how they learned about the death of their loved ones, as well as the last moments of their loved one's life and the impossibility of visiting them in the hospital. Several participants pointed out the loneliness of the dying loved one, revealing a sense of anger and helplessness.

Group 2 also features two categories. The first, *End‐of‐life context* (present also in Group 1), concerns the elements that circumscribed the loss, such as the relationship with the medical staff and the patient's clinical history.

The second corresponds to a category present exclusively in Group 2 and centred on *COVID‐19*: the excerpts reveal the participants' experiences, tension, fear, and their management method, on an empathic and communicative level, of the medical staff following the contagiousness of the deceased.

#### The subjective grief experience

For Group 1, this category concerns the *experience* linked to the perception and consequences of losing a loved one and missing them: there are temporal elements, feelings and a personal vision of one's grief.

Concerning Group 2, two categories are also present: *Grief experience*, which concerns the perception and consequences of loss and missing the person, and *Emotions of loss*. Sadness is the most prevalent emotion in the participants' speech, with many expressing that they continue to cry despite the passing of time and discussing how much they miss their loved ones. Additionally, some participants report a feeling of anger:

So much sadness, so much anger. Right now, I think we can talk about a phase of depression. I don't know if it's an emotion, but I have many tears, many tears, and sadness.

#### Social support

For Group 1, *social support* is perceived even during restrictions and isolation. However, for Group 2, this topic corresponds to the emergence of an ambivalent perception by the participants: some of them feel supported and able to express their feelings and difficulties, while others feel that they are not fully understood, aware that each bereavement and reaction to loss is personal.

#### Other bereavements

*Other bereavement* is a category that is present exclusively in Group 1. Although these participants chose to complete the questionnaire thinking exclusively of one person during the interviews, different losses that had occurred since the beginning of the pandemic emerged. These extracts describe traumatic events, with initial grief about the manner of death, the funeral rites performed and the restrictions.

### Thematic analysis and comparison of Groups 1 and 2 in T1

#### End‐of‐life context and health restrictions

In T1, these categories are present simultaneously in the same class for both groups. Concerning Group 1, *End‐of‐life context* describes more the experiences of illness, hospitalisation and death, events perceived as brutal, sudden and unexpected:

He had some heart problems but was very well taken care of medically. So he wasn't in a serious condition that day. Then suddenly, within three hours, his condition worsened. His doctor came to see him immediately.

The second category, *Health restrictions*, emerged exclusively at T1 for both groups. It highlights how the pandemic altered treatment, leading to a lack of communication and closeness with the hospitalised patient, which in turn gave rise to feelings of helplessness, anger and abandonment.

As for Group 1, in the *End‐of‐life context*, Group 2 refers to how the rules imposed for the spread of the virus changed contact and communication with the patient.

In general, this subject interrogates the topics of the second wave, the vaccine, COVID‐19, that is, everything that continued to generate worries and anxieties during that period.

#### Categories covering funeral restrictions

Class 2 of Group 1 includes *Funeral restrictions* and *Other arrangements*, that is, the ceremonies that bereaved families could organise only months after the death.

The first category includes examples of how the restrictions affected the traditional mourning customs: in many cases, it was not possible to carry out the wishes that the deceased had expressed for his or her own funeral, or approach and dress the body, or respect the traditional timeframes for the funeral.

From the accounts emerge a perception of a lack of respect towards the deceased, their desires, their body, and feelings of anger and disbelief.

As in T0, Group 2 had a *Funerary Rites* category. The extracts seem to highlight how the participants are satisfied with the rites they were able to perform, even if not all the desired people could be present:

My paternal grandparents weren't believers, so we didn't do much. I admit that on his birthday, August 3rd, I still thought about him, saying: he would have been 87 years old. It's one of the little things you tell yourself: his birthday.

Furthermore, the third and final class of Group 2 concerns the body. In particular, the participants describe the moments following the death concerning the possibility or their need for *Seeing or touching the body*. No particular references to health restrictions emerge from the speech, but rather the personal choice of the participants not to go to see the body or touch it.

#### The subjective grief experience

In Class 3 of Group 1, there is the category of *Current grief*, that is, the perception of one's grief in the present and how this has changed over the months. All the participants agree that they have noticed an evolution in their emotions, whilst also making referencing elements of lingering/persistent sadness and anger.

Additionally, Class 3 includes stories about *Memories of the deceased*, a category not present in T0 or in Group 2. This class represents individual and family strategies for coping with grief, including the sharing of photos and memories with loved ones to avoid facing their grief alone.

In Group 2, Class 1 similarly addresses *Current grief*, but with an emphasis on painful memories and events that evoke sadness and melancholy.

#### Social support

As in T0, also in T1, the last class of Group 1 corresponds to *Social support*, perceived as constant even after several months. However, concerning Group 2, in T1, there are no references to the support perceived by friends and family.

#### Social media

With the categories of *Current grief* and *Funeral rites* in Class 1 of Group 2, there is also that of *Social media*, which occurs exclusively in Group 2. Participants talked about both the support found through social media, the connection tools used particularly during the pandemic, and their function in announcing the deaths. Some of them have transformed the profiles of the deceased into a memorialised accounts:

I don't know how to say it, I've turned his Facebook page into a memory page, but it's the only digital thing I've done for my father's death.

For others, their goal was to find emotional support. Social media allowed them to share their grief with people who were going through the same thing, which reinforced their feelings of understanding and empathy:

I subscribed to these pages and it's true that many people talk about it, I also lost my mother, and they talk about it there.

## DISCUSSION

This study aimed to shed light on the unique challenges faced by individuals who lost a loved one in France during the initial lockdown period (Group 1), comparing their experiences with those bereaved during other periods (Group 2).

The findings indicate distinct differences between the two groups. Group 1 reported a broader spectrum of negative emotions, including feelings of helplessness, abandonment and anger (see Appendices S1–S3).

In T0, the discourse centred on *funeral restrictions* (Group 1, Class 1) highlights how Group 1 failed to understand any causal link between the pandemic and death. The absence of traditional funeral rituals and social support exacerbated their sense of isolation and emotional disconnection, intensifying the grieving process and acceptance of loss.

Conversely, those in Group 2 were more resilient and faced fewer restrictions, allowing them to partake in more funeral rites, which lessened the impact on their grieving process. Group 2 demonstrated predominantly feelings of sadness and an emotional experience that suggests a more secure and contained environment, allowing the effects of loss and vulnerability to be expressed. Concerns over the uncertainty of the pandemic and challenges in communication with the patient characterised their experiences. Notably, contextual and organisational factors emerged as influential determinants of grief experiences.

Participants in Group 1 recounted narratives overshadowed by the restrictive health context, impeding their ability to bid proper farewells and navigate the mourning process effectively. In contrast, those in Group 2 recalled a more flexible environment. They express their resistance to *Touching or seeing the body*—an impossible scenario, though, for those who lost someone during lockdown. The latter spoke about how the restrictions changed the funeral rites, expressing a sense of helplessness, nostalgia, disbelief, and anger at how the professionals treated the coffin and at not being able to say goodbye to their loved ones (*Funeral restrictions*). In contrast, participants in Group 2, who did not experience the loss in lockdown, did not discuss the restrictions but rather reported elements significant to the ritual, such as the service, flowers and the church. These 20 participants were able to share their emotions and mourn in a family environment, experiencing and expressing their pain and sadness according to their traditions and needs.

Furthermore, Group 1 described the entire period following the loss in a traumatic manner, including further grieving (*Multiple bereavements*, a category present exclusively in this group). This aspect could be further confirmation of the fact that the lockdown and the absence of rituals aggravated the grieving process, resulting in a psychological inability to separate the various losses.

Considering the demographic characteristics of the deceased is crucial in understanding the influence of age on grieving. With an average age of around 70, seniors faced higher mortality rates during the pandemic. Specifically, Group 1 experienced a higher proportion of COVID‐19‐related deaths (36%) compared to Group 2 (15%). Elderly individuals, more vulnerable to severe outcomes, likely faced increased mortality rates and fears of their mortality, which may have intensified their bereavement experiences.

In contrast, Group 2's younger age (average 46) suggests a greater inclination towards utilising social media for solace and connection, reflecting broader social media usage trends among younger demographics (Zote, 2024). Conversely, older participants in Group 1 likely relied more on traditional support disrupted by lockdown measures.

Thus, understanding the interplay between age, mortality rates, and grief experiences provides insight into the multifaceted nature of bereavement during the pandemic.

In addition to the primary focus on lockdown versus no lockdown, this study acknowledges the potential influence of other variables such as gender, relationship to the deceased, living alone, and the suddenness of death. Although the ALCESTE analysis did not specifically highlight these variables, they are crucial factors that could shape the grieving experience. For instance, gender differences in emotional expression, the closeness of the relationship to the deceased, the impact of living alone on perceived social support, and the shock of sudden deaths are all aspects that warrant further investigation. Future research should aim to explore these variables in more detail to provide a comprehensive understanding of the multifaceted nature of grief during the pandemic.

## CONCLUSION

The findings of this study offer crucial insight into the experiences of individuals who suffered loss during the initial French lockdown. They underscore the profound impact of isolation and restrictions on the grieving process, highlighting the heightened intensity and prolonged nature of grief among those bereaved during the strict lockdown period. There is a pressing need for enhanced support and communication between families and healthcare professionals to alleviate confusion and distress among the bereaved (Dew et al., 2022; Hanna et al., 2021).

A comparative analysis between Group 1 and Group 2 reveals significant differences in their grieving experiences, emphasising the role of pandemic‐related factors and the absence of customary rites in shaping the grieving process.

While these findings cannot be generalised to the entire population, they provide more information and insight into other people's experiences in similar circumstances.

No significant needs emerged regarding how the restrictions were announced; however, it is confirmed that having the opportunity to say goodbye to one's loved one is a fundamental need, along with respecting the needs at the end of life and post‐mortem, both of the dying and the grieving person (Bacqué & Hanus, 2023; Cherblanc, Zech, et al., 2022; Dew et al., 2022).

These results emphasise the need to preserve social ties and support networks during periods of mourning, highlighting the importance of both individual and group interventions. It is crucial to promote public policies that facilitate access to support services and resources, thus fostering resilience and adaptation mechanisms among grieving individuals.

Furthermore, respecting cultural and personal preferences during end‐of‐life and post‐mortem processes is essential. Policies should prioritise dignity and honour diverse cultural practices, ensuring that individuals could bid farewell to their loved ones in accordance with their beliefs and traditions. By improving support systems for grieving individuals, it is possible to better address the varied needs of mourners during times of crisis.

In conclusion, our study underscores the significance of compassion, support, and cultural sensitivity in facilitating the grieving process and ensuring the well‐being of individuals and families affected by loss. Through concerted efforts to prioritise the needs of the bereaved, we can navigate the complexities of grief with resilience and empathy.

## LIMITATIONS

This study has some limitations. Firstly, the participants' groups are not homogeneous, with 11 participants in Group 1 and 20 in Group 2, which may affect the comparability of the findings. Additionally, the recruitment of participants was not standardised concerning the cause or the time of death, with the only criterion being that the death occurred after March 2020. Finally, the selection of participants and the analysis of the interviews did not consider other possible criteria, such as the cause of death.

## COMPLIANCE WITH ETHICAL STANDARDS

All procedures performed in studies involving human participants were following the ethical standards of the institutional research committee at University of Strasbourg (Unistra/CER/2021‐16) and with the 1964 Helsinki Declaration and its later amendments or comparable ethical standards. Informed consent was obtained from all individual adult participants in the study.

## Supporting information


**Appendix S1.** The sentences selected and analysed by ALCESTE for Groups 1 and Group 2 in T0.
**Appendix S2.** The sentences selected and analysed by ALCESTE for Groups 1 and Group 2 in T1.
**Appendix S3.** Example in English of the table that emerged following the analysis of ALCESTE with respect to Group 1 in T0.
