# Supplementary material for: Impact of French lockdowns on bereavement experiences: Insight from ALCESTE analysis revealing psychological resilience and distinct grief dynamics amidst COVID‐19
Source: Int J Psychol. 2024 Nov 19;60(1):e13267. doi: 10.1002/ijop.13267 (PMC11628357; doi:10.1002/ijop.13267)
Supplement: Supplementary file 1 — Appendix S1. The sentences selected and analysed by ALCESTE for Groups 1 and Group 2 in T0. Appendix S2. The sentences selected and analysed by ALCESTE for Groups 1 and Group 2 in T1. Appendix S3. Example in English of the table that emerged following the analysis of ALCESTE with respect to Group 1 in T0. [file IJOP-60-e13267-s001.docx]

**Appendices**

Appendix A. The sentences selected and analyzed by ALCESTE for Groups 1 and Group 2 in T0.

| **Group 1 - T0** | | | **Group 2 - T0** | | |
| --- | --- | --- | --- | --- | --- |
| **Class** | **Theme** | ***Verbatim*** | **Class** | **Theme** | ***Verbatim*** |
| **Class 1** | Funeral restrictions | *They closed the bag. They closed the bag. They put it in a coffin and stored it in the morgue, at the hospital. We contacted the funeral home but there were already too many bodies and a queue for caskets. So we had to wait until they had availability in the funeral home.* | **Class 1** | End of life context | *He had heart problems, kidney problems, he had many health problems. I called the emergency room and they admitted him for 2 or 3 days, then he was fine and his fever went down. He was released from the hospital on Sunday.* |
|  |  |  |  |  | *I am having jaw surgery and my mother's oncologist calls me saying her condition has worsened and they are transferring her to palliative care.* |
|  |  | *There was no preparation of the body, so we had the impression that he was put in a garbage bag like a plague victim and then put in a coffin. A few days later, we found ourselves in front of a closed coffin. We put a photo on the coffin so we would know it was him.* |  | COVID-19 | *She came to help me wash him and also saw that he was more and more tired. I called his doctor and for me, it was psychological, because I knew he had COVID. She wanted to come in the afternoon and I insisted because I saw he wasn't well.* |
|  |  |  |  |  | *In the afternoon I went to the hospital, in the COVID ward, to see mom from a window. They dressed me up as a COVID nurse and I was finally able to enter the room, but officially it wasn't allowed.* |
| **Class 2** | Medical and health care team | *He got sick, he had pneumonia so I went to visit him in the hospital. I helped him and one of the nurses, during a visit, asked me if I could stay with him because he would have a rather difficult exam to pass.* | **Class 2** | Grief experience | *My life resumed normally, I never stopped, and I didn't stop working. I normally resumed my work the day after the mourning, the day after the burial.* |
|  |  | *I told him, "Look, he looks tired," and then I asked him if he could come tonight or tomorrow, that everything would be fine but there was no urgency. Luckily the nurse came back and she found him with a fever and in failure, so the doctor came right away.* |  |  | *I tried to occupy my mind as much as possible. Normally I am a person who likes to be alone and who likes to be quiet at home and there I a little prevented myself from feeling all the emotions that there were* |
|  | End of life context | *He was contaminated with COVID from another patient. He was contaminated during hospitalization in addition to untreated cancer. And the great tragedy is that, despite doing everything, I got in touch with A.K., the oncologist, and he said: "seeing what you're telling me, ask for palliative care to be activated for your father".* |  | Emotions of loss | *So much sadness, so much anger. Right now, I think we can talk about a phase of depression. I don't know if it's classified as an emotion, but I have many tears, many tears, and sadness.* |
|  |  |  |  |  | *After the death and burial and everything, I went back to work, and then there were hard times, times when I wanted to cry and put myself in a corner.* |
|  |  | *He couldn't catch his breath, we didn't have good news from the hospital but we had some hope. And then, on Monday or Sunday, the hospital notified my sister that they were suspecting a pulmonary embolism.* |  | Social support | *I think I live with it quite well and, in quotes, have a strong family unit. If I want to talk, my parents are open to discussion, to cheer me up if needed.* |
|  |  |  |  |  | *It's always complicated because not everyone experiences grief the same way and people who haven't lost a parent have a hard time understanding. They try their best, but they don't understand.* |
| **Class 3** | Grief experience | *I feel the isolation in this pain. Although it has progressed, I can tell you that I have been feeling this way for over a year now. I feel I'm a little sensitive when I talk to you, but it's normal, that is, I talk about my... But in the meantime, there are so many... I really feel that the mourning is progressing.* | **Class 3** | Funeral rites | *We had both a religious ceremony and a cemetery ceremony. She had a beautiful ceremony, everyone told us. I forced mysel and wrote a text, I struggled with myself to read it out.* |
|  |  | *I am progressing in my pain. I was accompanied by an amazing person who does bereavement counseling. There are things I can do... It's been a year but there's still this time in the hospital that... I can't get over it, remains a moment of anger.* |  |  | *There was a religious ceremony for the funeral service, otherwise, we also put flowers, and a plaque with the little children. We bought flowers for the burial.* |
| **Class 4** | Social support | *It's all a bit wired, because until May, we were locked up at home as well, so it was a long-distance support. There are friends who came forward and then people who...* | **Class 4** |  |  |
|  |  | *I have my sister too, I have a good relationship with her. I've had support from friends. There are a few people who paided attention to what happened.* |  |  |  |
|  | Multiple bereavements | *Before his death I lost a very close friend by a car accident. It was in December 2019, and then he was the biggest shock. Since then I have lost quite a few acquaintances, older people, but also a brother-in-law of my sister, he was 64 years old.* |  |  |  |
|  |  | *He died in an car accident so I was quite affected, and then there was also my brother-in-law. Then I did a lot of funerals because I have three close friends who lost one of their parents, so...* |  |  |  |

Appendix B. The sentences selected and analyzed by ALCESTE for Groups 1 and Group 2 in T1.

| **Group 1 - T1** | | | **Group 2 - T1** | | |
| --- | --- | --- | --- | --- | --- |
| **Class** | **Theme** | ***Verbatim*** | **Class** | **Theme** | ***Verbatim*** |
| **Class 1** | End of life context | *He had some heart problems, but was very well taken care of medically. So he wasn't in a serious condition that day. Then suddenly, within three hours, his condition worsened. His doctor came to see him immediately.* | **Class 1** | Social networks | *I don't know how to say it, I've turned his Facebook page into a memory page, but it's the only digital thing I've done for my father's death.* |
|  |  | *The doctor immediately called the ambulance and he was immediately admitted, it was a Saturday, yes, Saturday, March 28, at 12:00. He received respiratory assistance, and then when he got to the hospital, he was intubated very quickly, very quickly.* |  |  | *I subscribed to these pages and it's true that many people talk about it, I also lost my mother, and they talk about it there.* |
|  | Health restrictions (hospital context) | *The doctor authorized us to visit him but he was alone, with no one, without even a psychologist. We saw our father completely emaciated. We weren't allowed near him as we were in a COVID unit and there were no masks. We wear masks only on the first day.* |  | Current grief | *It's something my grandfather might have said or done or his way of thinking. I don't talk about him often, but personally, I think about him regularly and without sadness.* |
|  |  | *So, all of a sudden, the dead from COVID were no longer being treated, even though there was no more respiratory function. Embalming treatments were forbidden. That, we can understand because the fluids were contaminants.* |  |  | *I've been through some pretty gross stuff. And there are a lot of traumatic memories that are coming back now, a year later.* |
| **Class 2** | Funeral restrictions | *He got sick, he had pneumonia so I went to visit him in the hospital. I helped him and one of the nurses, during a visit, asked me if I could stay with him because he would have a rather difficult exam to pass. He was put in a bag and then left for several days in the hospital basement, in the equivalent of a cold room, as the funeral home was overflowing.* | **Class 2** | End of life context | *There was a misdiagnosis, the doctor said it was a mild form while on oxygen at home* |
|  | Other arrangements | *On September, 9th, she arranged a ceremony, like a burial, only there was no coffin.* |  |  | *His condition was made worse by the vaccine. I talked about it with my doctor, he told me that I shouldn't get this idea into my head, that unfortunately a metastatic tumor like his cannot be cured.* |
|  |  | *So we went and put flowers on the grave and that was it. Also, since my sister's birthday is on the 26th of September, we had a party on Sunday the 31st, the day before All Saints' Day, so...* |  | Health restrictions | *After four days of hospitalization in the Covid ward, they told him: "Sir, you are taking away a bed from us for nothing." These are the words of the doctor of the COVID ward. We will take you home and you will keep the barrier gestures with your wife.* |
|  |  |  |  |  | *I too have health problems, I couldn't catch COVID too, so barrier gestures, such as visors, masks, gloves, etc* |
| **Class 3** | Current grief | *Today I think I experienced a shock wave, I experienced a shock wave, I experienced something so violent, so violent, I truly perceived this end of life without support in this way.* | **Class 3** | Seeing or touching the body | *So the coffin was closed, I didn't see it at all. Mmy other son came down with the funeral home workers to pick him up.* |
|  |  | *When you are grieving, you are trapped in your mourning, you are not well. And yes, there were moments when… it happens often… when you are sad… I didn't feel good anywhere, this feeling was so bad that I wanted to run away.* |  |  | *The doctor told me: "Do you want to see him?". I said :"Yes", but I didn't dare touch him. He was in our room and I stood by the door.* |

Appendix C. Example in English of the table that emerged following the analysis of ALCESTE with respect to Group 1 in T0.

| **Class 1** | **Class 2** | **Class 3** | **Class 4** |
| --- | --- | --- | --- |
| Coffin | Doctor | Thing | Brother |
| Funeral | Morning | Life | Anniversary |
| Home | Nurse | Moment | Friend |
| Ash | Hospital+ | Grief | Burial |
| Body | Hospital | Love | Sister |
| Funera+ | Call | Say | Brother in law |
| Ceremony | Charge | Feel | Accident |
| Casket | Father | Angry | Cousin |
| Crema | Psychologist | Lack | December |
| Close | Cure | Side | Close |
| Put | Monday | Wish | Idea |
| Scatter | Sick | Child | January |
| Bag | Medici+ | Work | Pleasure |
| Urn | Cancer | Time | Good |
| Vault | Unit | Candle | Date |
| Room | Urgent | Talk | Car |
| Morgue | Condition | Think | September |
| Object | Sedat | Internet | Same |
